# Association between Modified Body Mass Index and 30-Day and 1-Year Mortality after Intensive Care Unit Admission: A Retrospective Cohort Study

**DOI:** 10.3390/jcm7040081

**Published:** 2018-04-13

**Authors:** Tak Kyu Oh, Jaebong Lee, Yeon Joo Lee, Jung-Won Hwang, Sang-Hwan Do, Young-Tae Jeon, In-Ae Song

**Affiliations:** 1 Department of Anesthesiology and Pain Medicine, Seoul National University Bundang Hospital, 166, Gumi-ro, Bundang-gu, Seongnam 463-707, Korea; airohtak@hotmail.com (T.K.O.); jungwon@snubh.org (J.-W.H.); shdo@snu.ac.kr (S.-H.D.); ytjeon@snubh.org (Y.-T.J.); 2 Medical Research Collaborating Center, Seoul National University Bundang Hospital, 166, Gumi-ro, Bundang-gu, Seongnam 463-707, Korea; 02113@snubh.org; 3 Division of Pulmonary and Critical Care Medicine, Department of Internal Medicine, Seoul National University Bundang Hospital, 166, Gumi-ro, Bundang-gu, Seongnam 463-707, Korea; yjlee1117@snubh.org

**Keywords:** albumin, body mass index, critical care, intensive care unit, mortality

## Abstract

Because conventional body mass index (cBMI) does not reflect fluid accumulation, modified BMI (mBMI, serum albumin multiplied by cBMI) is a more accurate measure of malnutrition status. This study aimed to determine whether mortality after intensive care unit (ICU) admission was associated with cBMI, mBMI, and/or serum albumin levels. The medical records of patients who were admitted to a tertiary hospital ICU between 1 January 2012 and 31 July 2016 were retrospectively reviewed. In total, 6169 ICU-admitted patients were included in the analyses. Multivariate Cox regression analyses revealed that low cBMI, mBMI and albumin level were significantly associated with 30-day and 1-year mortality after ICU admission (hazard ratio < 1.0, *p* < 0.05). The adjusted area under the curve (AUC) of mBMI for 1-year mortality was significantly higher than that of cBMI (*p* < 0.001), but not significantly different from that of albumin level (*p* = 0.098). Low values of mBMI, cBMI and albumin were independently associated with 30-day and 1-year mortality after ICU admission. Combining cBMI and albumin (mBMI) did not increase the validity of the AUC of albumin for 1-year mortality after ICU admission. Our study showed that serum albumin alone, rather than mBMI (combining cBMI), is recommended in predicting mortality among ICU patients.

## 1. Introduction

Malnutrition is related to prognosis and mortality among patients admitted to all types of intensive care units (ICUs) [1], including medical and surgical ICUs [2,3]. Thus, various methods have been developed to evaluate nutritional status and predict prognosis among these patients, with conventional BMI (cBMI) and serum albumin levels being widely used in this setting [4,5]. A multicenter prospective cohort study revealed that low cBMI was associated with increased mortality among ICU-admitted patients [6], and a retrospective observational study revealed that low serum albumin levels at ICU admission were associated with patient prognosis [7].

Although serum albumin and cBMI may help identify malnutrition at ICU admission, their efficacy has been questioned [8,9]. For example, although cBMI reflects obesity and the patient’s general physical status, it does not reflect fluid balance, such as fluid accumulation or dehydration. This issue is particularly relevant after liver transplantation when patients are highly susceptible to ascites [10,11]. In contrast, serum albumin levels reflect fluid balance and ascites, but do not provide accurate information regarding general physical status, such as obesity or simple weight loss. Thus, the modified BMI (mBMI) was developed to overcome these limitations [10,11], as it combines serum albumin levels and cBMI, and is accurate for assessing patients undergoing liver transplantation [8,9,10,11]. Thus, given the importance of malnutrition in the ICU [1], mBMI may be a useful prognostic tool in this ICU setting. Therefore, the present study aimed to determine whether 30-day and 1-year mortality after ICU admission are associated with mBMI, cBMI, and/or serum albumin concentration.

## 2. Materials and Methods

This retrospective observational study was performed with the approval of the institutional review board of our hospital (B-1706/402-106). Adult patients (≥19 years old) were considered eligible if they were admitted to the ICU between 1 January 2012 and 31 July 2016. Only the final admission was considered in cases with multiple admissions, and cases with inaccurate or incomplete medical records were excluded. As of August 2017, our hospital is a 1360-bed capacity tertiary care hospital with 111 ICU beds in 5 ICUs (medical, surgical, neurological, emergency I, and emergency II). Since 2003, our hospital has managed all medical records using an electronic medical record system.

### 2.1. Definition of mBMI 

The mBMI values were calculated as cBMI (kg/m^2^) × serum albumin (g/L) based on the methods of previous studies [8,9]. Height (cm) and weight (kg) were measured at ICU admission, while the serum albumin level was selected from the test that was closest to the ICU admission date. In our hospital, laboratory tests for serum albumin are routinely performed for most ICU patients within 1 day after ICU admission or immediately before ICU admission.

### 2.2. Data Collection and Outcome

The retrospectively collected data included sex, age, height, weight, cBMI, mBMI, length of hospital and ICU stay, Acute Physiology and Chronic Health Evaluation II score, medical history (hypertension, diabetes mellitus, ischemic heart disease, and/or liver disease), diagnosis of cancer, blood laboratory test results, and date of death.

Only laboratory results obtained immediately after ICU admission were used. A history of ischemic heart disease was defined as diagnoses ranging from stable angina to myocardial infarction. Dates of death were determined with approval from the Korean Ministry of the Interior and Safety, and the analyses extended until 1 August 2017. All data were collected by members of the medical informatics team, who were blinded to the study objectives, and the main researchers were blinded to the data until the final statistical outcomes were derived. 

The primary endpoint was the association between mBMI at ICU admission with 30-day and 1-year mortality after ICU admission, while the secondary endpoint was the comparison of mBMI with cBMI and albumin levels.

### 2.3. Statistical Analysis

The patients’ characteristics were presented as number (%) or median [Interquartile range]. After testing for normality of the data of the continuous variables according to 30-day mortality and 1-year mortality using the Kolmogorov–Smirnov test, continuous variables were compared using the Mann–Whitney *U* test, while categorical variables were compared using the chi-square test. We used restricted cubic splines to illustrate the log odds of 1-year mortality according to the three prognostic variables (cBMI, mBMI and albumin). Univariate Cox logistic regression analyses were initially performed, and variables with *p*-values <0.1 were subsequently included in the multivariate Cox logistic regression analyses to determine whether the prognostic variables were associated with 30-day and 1-year mortality after ICU admission. In this Cox regression model, we divided the mBMI, cBMI and albumin values into quartiles to compare hazard ratios (HRs) with 95% confidence intervals (CIs) for 30-day and 1-year mortality after ICU admission. 

Finally, receiver operating characteristic (ROC) curve analysis was used to evaluate the prognostic values of the variables, and covariate-adjusted ROC curve analyses were used to determine the adjusted areas under the curves (AUCs), which were compared using Delong’s test. All analyses were performed using R software (version 3.3.2; http://www.R-project.org), and differences were considered statistically significant at *p*-values of <0.05.

## 3. Results

Between 1 January 2012 and 31 July 2016, 9354 patients were admitted to the ICUs of our hospital. After excluding 2324 cases with multiple admissions, and 861 patients with missing or inaccurate data regarding BMI or serum albumin from the ICU admission, the final analyses included data from 6169 patients. These patients’ demographic and clinical characteristics are shown in Table 1.

### 3.1. Relationships between 30-Day Mortality, 1-Year Mortality and the Prognostic Variables (cBMI, mBMI, and Albumin)

**Figure 1:**
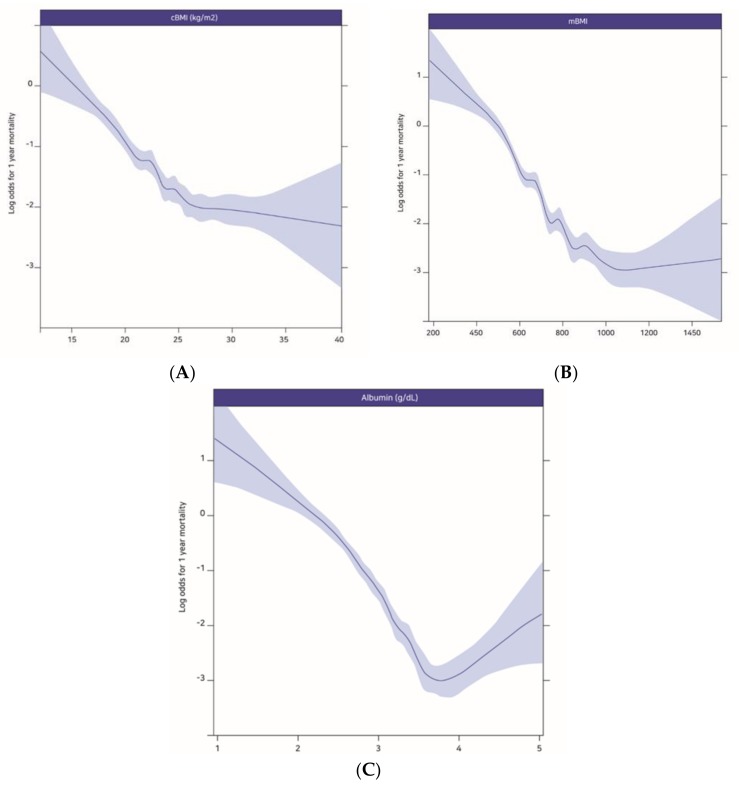
The log odds for 1-year mortality plotted against the changes in cBMI (**A**), mBMI (**B**) and albumin (**C**). cBMI: conventional BMI, mBMI: modified BMI.

Figure 1 shows the relationships between 1-year mortality after ICU admission and cBMI (A), mBMI (B), and albumin (C). These relationships were illustrated using restricted cubic splines, and the results indicated that decreasing values of the prognostic variables were associated with increasing 1-year mortality after ICU admission.

### 3.2. Risks of 30-Day and 1-Year Mortality after ICU Admission According to cBMI, mBMI, and Albumin Values

Appendix A Table A1 and Table A2 shows the results of the univariate Cox regression analyses of 30-day and 1-year mortality after ICU admission among all patients. Table 2 shows the results of the multivariate Cox regression analyses, which revealed that 30-day mortality was independently associated with cBMI (HR of Q3 (versus Q1): 0.706, 95% CI: 0.541–0.922, *p* = 0.010), mBMI (HR of Q2 (versus Q1): 0.556, 95% CI: 0.449–0.712; HR of Q3 (versus Q1): 0.296, 95% CI: 0.216–0.404; HR of Q4 (versus Q1): 0.223, 95% CI: 0.155–0.320; all *p* < 0.001) and serum albumin (HR of Q2 (versus Q1): 0.385, 95% CI: 0.306–0.485; HR of Q3 (versus Q1): 0.182, 95% CI: 0.124–0.268; HR of Q4 (versus Q1): 0.131, 95% CI: 0.081–0.210; all *p* < 0.001).

Table 3 shows the results of the multivariate Cox regression analyses, which revealed that 1-year mortality was independently associated with cBMI (HR of Q2 (versus Q1): 0.715, 95% CI: 0.618–0.828; HR of Q3 (versus Q1): 0.525, 95% CI: 0.445–0.619; HR of Q4 (versus Q1): 0.471, 95% CI: 0.395–0.561; all *p* < 0.001), mBMI (HR of Q2 (versus Q1): 0.529, 95% CI: 0.459–0.611; HR of Q3 (versus Q1): 0.302, 95% CI: 0.252–0.363; HR of Q4 (versus Q1): 0.207, 95% CI: 0.165–0.258; all *p* < 0.001) and serum albumin (HR of Q2 (versus Q1): 0.509, 95% CI: 0.444–0.584; HR of Q3 (versus Q1): 0.264, 95% CI: 0.214–0.324; HR of Q4 (versus Q1): 0.221, 95% CI: 0.172–0.282; all *p* < 0.001).

### 3.3. Covariate-Adjusted ROC Analysis of 1-Year Mortality According to the Prognostic Variables (cBMI, mBMI, and Albumin) 

**Figure 2:**
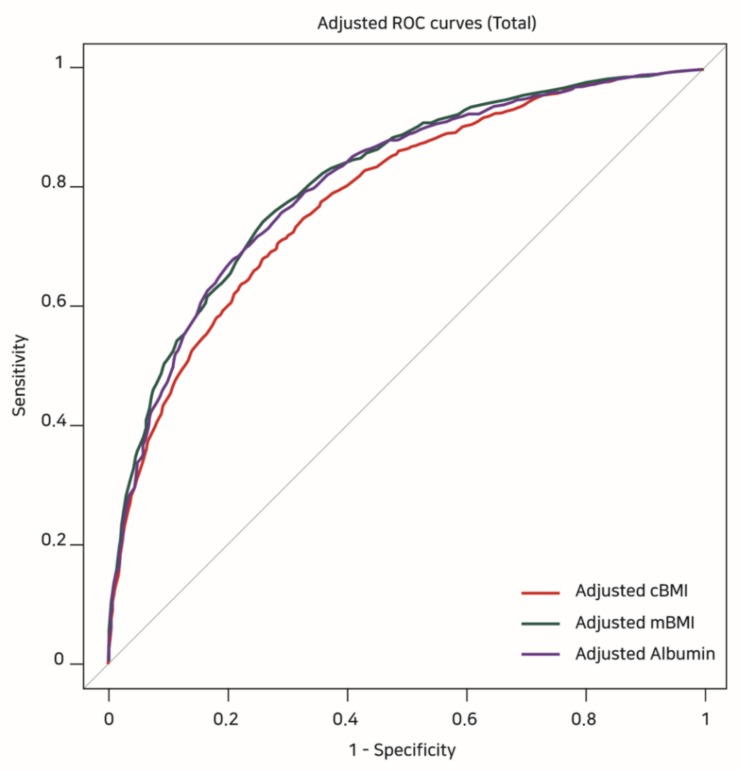
Covariate-adjusted ROC curves showing the risk of 1-year mortality according to cBMI, mBMI, and albumin values. ROC: receiver operating characteristic, cBMI: conventional BMI, mBMI: modified BMI.

Table 4 shows the results of the covariate-adjusted ROC analyses. The highest adjusted AUC was observed for mBMI (0.819, 95% CI: 0.800–0.827), which was followed by albumin (0.808, 95% CI: 0.794–0.822) and cBMI (0.784, 95% CI: 0.769–0.798) (Figure 2). However, comparing the adjusted AUCs from Delong’s test, the adjusted AUCs of mBMI and albumin were not significantly different (Z = −1.653, *p* = 0.098), while the adjusted AUCs of mBMI and albumin were higher than that of cBMI (adjusted AUC of cBMI versus mBMI: Z = 8.216, *p* < 0.001, adjusted AUC of cBMI versus albumin: Z = 4.536, *p* < 0.001).

## 4. Discussion

The present study showed that cBMI, mBMI and albumin values at ICU admission were associated with 30-day and 1-year mortality after ICU admission. Although mBMI had a higher AUC of association with 1-year mortality after ICU admission than cBMI before and after adjusting for covariates, mBMI did not show a significantly higher AUC than serum albumin. This means that combining cBMI and serum albumin level did not increase the validity of the AUC of albumin for mortality after ICU admission. This is in accordance with the results of previously published reports [12].

Patients admitted to the ICU have increased incidence of sepsis [13] and acute respiratory distress syndrome [14], or are more likely to be elderly patients with other severe illnesses [15]. In this context, malnutrition is linked to immune function, and various studies have examined nutritional support for enhancing the immune functions of patients in the ICU [16,17]. In this regard, serum albumin and cBMI are considered important factors associated with malnutrition among patients [4,5]. Moreover, as cBMI and albumin are closely associated with pressure ulcer development in the ICU [18,19], it is possible that mBMI may predict pressure ulcer development in the ICU, although additional studies are needed to test this hypothesis. Based on this, we hypothesized that using mBMI, which simultaneously reflects cBMI and serum albumin, would better show the association with mortality after ICU admission than cBMI or serum albumin alone. However, combining cBMI and albumin did not increase the validity of the AUC of albumin in association with 1-year mortality as we reported in our previous study for general surgical patients [12].

The HRs of 1-year mortality for cBMI and mBMI exhibited an interesting pattern, with low cBMI or mBMI values associated with sharply increased risks of 1-year mortality, while elevated cBMI or mBMI values were not associated with large increases in the risk of 1-year mortality. Previous studies have indicated that obesity is an independent risk factor for mortality among patients in the ICU [20], while others have indicated that overweight or obese status can protect against mortality in this setting [21]. Furthermore, another study has indicated that high cBMI was not related to mortality in the ICU, although obesity based on sagittal abdominal diameter was an independent risk factor for mortality [22]. Thus, the relationship between obesity/cBMI and mortality among patients admitted to the ICU remains controversial. However, it is important to consider the prevalences of obesity when comparing the results from American and Korean studies. In the U.S., 34.9% of adults had a cBMI of ≥30 kg/m^2^ during 2011–2012 [23], compared to a Korean prevalence of only 4.1% for a cBMI of ≥30 kg/m^2^ in 2007 [24]. Therefore, national and regional differences must be considered when evaluating the effects of high cBMI or mBMI on patients in the ICU.

Unlike albumin or cBMI, which have widely accepted normal ranges, there is no clear standard for categorizing mBMI, as only a few studies have examined this issue. Tanaka et al. have classified mBMI into six groups (<600, 600–800, 800–1000, 1000–1200, 1200–1400, and >1400) [9], while Suhr et al. have reported a high mortality rate after liver transplantation among patients with an mBMI of <600 [10]. In our study, we divided the mBMI values into quartiles to compare HRs (Q1 < 636.0, 636.0 ≤ Q2 ≤ 759.0, 759.0 < Q3 ≤ 884.0, and Q4 > 884.0) with cBMI and albumin. Therefore, these cut-off values of mBMI can be useful references for previous studies [9,10].

The present study has several limitations. First, the retrospective design is associated with risks of selection bias. However we tried to minimize selection bias; data collection was conducted by medical records technicians, who were blinded to the study objectives. Second, the evaluation of patients from a single center precludes generalization to other centers or populations. Third, the relatively low prevalence of obesity in Korea may also preclude generalization to other populations. Fourth, considering that laboratory tests for serum albumin concentration are not routinely performed in other settings outside the ICU, the prognostic value of mBMI might have limited utility in clinical practice. Lastly, we did not have access to detailed mortality data and the deaths could have been unrelated to the indications for ICU admission, although we can confirm that the dates of death were accurate based on data from the Korean government. 

The present study showed that low values of mBMI, cBMI, and albumin were independently associated with 30-day and 1-year mortality after ICU admission. While mBMI had higher prognostic value for association with 1-year mortality than cBMI, it was not significantly different in prognostic value from serum albumin level. Therefore, our study showed that serum albumin alone, rather than mBMI (adding cBMI), is recommended in predicting mortality among patients in the ICU.

## Figures and Tables

**Table 1:** Patients’ demographic and clinical characteristics.

Variable	*n*	%	Mean	SD	Min	Max
Age (year)	6169		63.4	15.6	18.0	100.0
Sex: Male	3719	60.3%				
Department: IM	1070	17.3%				
Postoperative Admission	4863	78.8%				
Height (cm)	6169		162.7	9.3	105.0	196.0
Weight (kg)	6169		64.2	12.4	24.1	128.9
cBMI (kg/m^2^)	6169		24.2	3.9	11.0	45.3
mBMI	6169		763.8	187.3	133.1	1791.0
Length of Hospital stay (day)	6169		26.4	43.8	1.0	1603.0
Length of ICU stay (day)	6169		4.3	8.3	1.0	266.0
APACHE II score	6169		20.8	8.0	0.0	57.0
History of Hypertension	523	8.5%				
History of DM	51	0.8%				
History of IHD	95	1.5%				
History of Liver Disease	44	0.7%				
Cancer	1334	21.6%				
Albumin (g/dL)	6169		3.1	0.5	0.7	7.3
BUN (mg/dL)	6169		18.4	13.4	1.0	173.0
Creatinine (mg/dL)	6169		1.0	1.1	0.1	17.1
AST (IU/L)	6169		165.6	1147.9	4.0	20,000.0
ALT (IU/L)	6169		68.4	336.5	4.0	11,328.0
Hemoglobin (g/dL)	6169		10.9	1.7	3.7	18.3
Platelet count (×1000/μL)	6169		179.2	93.7	2.0	1154.0
Postassium (mEq/L)	6169		4.0	0.5	2.3	8.9
Sodium (mEq/L)	6169		137.8	4.4	119.0	168.0
WBC count (×1000/μL)	6169		12.3	10.6	0.1	703.9

SD, standard deviation; IM, Internal Medicine; cBMI, conventional body mass index; mBMI, modified body mass index; ICU, intensive care unit; DM, diabetes mellitus; IHD, ischemic heart disease; CKD, chronic kidney disease; BUN, blood urea nitrogen; AST, aspartate aminotransferase; ALT, alanine aminotransferase; WBC, white blood cell.

**Table 2:** Multivariate Cox regression analysis for 30-day mortality after ICU admission.

Variable	(+) cBMI		(+) mBMI		(+) Albumin	
	Hazard Ratio (95% CI)	*p*-Value	Hazard Ratio (95% CI)	*p*-Value	Hazard Ratio (95% CI)	*p*-Value
Sex: Male	1.201 (0.990–1.457)	0.063	1.162 (0.959–1.408)	0.125	1.150 (0.949–1.393)	0.154
Age (year)	1.009 (1.002–1.015)	0.011	1.005 (0.998–1.011)	0.147	1.005 (0.999–1.012)	0.122
Admission Department: IM	3.075 (2.493–3.792)	<0.001	2.352 (1.899–2.912)	<0.001	2.191 (1.775–2.705)	<0.001
Postoperative Admission	1.052 (0.847–1.308)	0.644	1.186 (0.958–1.469)	0.117	1.235 (0.999–1.526)	0.51
cBMI (kg/m^2^)						
Q1 < 21.7	1					
21.7 ≤ Q2 ≤ 24.1	0.906 (0.719–1.142)	0.403				
24.1 ≤ Q3 ≤ 26.6	0.706 (0.541–0.922)	0.010				
Q4 > 26.6	0.800 (0.617–1.039)	0.094				
mBMI						
Q1 < 636.0			1			
636.0 ≤ Q2 ≤ 759.0			0.556 (0.449–0.712)	<0.001		
759.0 ≤ Q3 ≤ 884.0			0.296 (0.216–0.404)	<0.001		
Q4 > 884.0			0.223 (0.155–0.320)	<0.001		
Albumin (g/dL)						
Q1 < 2.8					1	
2.8 ≤ Q2 ≤ 3.2					0.385 (0.306–0.485)	<0.001
3.2 ≤ Q3 ≤ 3.5					0.182 (0.124–0.268)	<0.001
Q4 > 3.5					0.131 (0.081–0.210)	<0.001
APACHE II score	1.090 (1.080–1.101)	<0.001	1.084 (1.074–1.095)	<0.001	1.080 (1.070–1.090)	<0.001
Hypertension	1.042 (0.753–1.443)	0.802	1.107 (0.803–1.526)	0.537	1.083 (0.788–1.489)	0.622
Diabetes Mellitus	0.767 (0.497–1.185)	0.232	0.771 (0.502–1.186)	0.236	0.698 (0.455–1.069)	0.098
History of IHD	0.942 (0.524–1.692)	0.842	0.936 (0.522–1.678)	0.824	0.969 (0.540–1.741)	0.916
Liver Disease	1.416 (0.665–3.016)	0.367	1.217 (0.572–2.591)	0.610	1.176 (0.553–2.501)	0.674
Cancer	1.424 (1.151–1.762)	0.001	1.340 (1.084–1.658)	0.007	1.349 (1.091–1.669)	0.006

cBMI, conventional body mass index; CI, confidence interval; mBMI, modified body mass index; IM, Internal Medicine; ICU, intensive care unit; APACHE II score, Acute Physiology and Chronic Health Evaluation II score; IHD, ischemic heart disease.

**Table 3:** Multivariate Cox regression analysis for one-year mortality after ICU admission.

Variable	(+) cBMI		(+) mBMI		(+) Albumin	
	Hazard Ratio (95% CI)	*p*-Value	Hazard Ratio (95% CI)	*p*-Value	Hazard Ratio (95% CI)	*p*-Value
Sex: Male	1.202 (1.062–1.360)	0.003	1.197 (1.058–1.353)	0.004	1.187 (1.050–1.342)	0.006
Age (year)	1.020 (1.016–1.025)	<0.001	1.017 (1.013–1.022)	<0.001	1.019 (1.014–1.023)	<0.001
Admission Department: IM	2.446 (2.132–2.805)	<0.001	2.014 (1.753–2.314)	<0.001	2.015 (1.755–2.313)	<0.001
Postoperative Admission	0.865 (0.754–0.993)	0.039	0.958 (0.836–1.098)	0.537	0.964 (0.841–1.105)	0.600
cBMI (kg/m^2^)						
Q1 < 21.7	1					
21.7 ≤ Q2 ≤ 24.1	0.715 (0.618–0.828)	<0.001				
24.1 ≤ Q3 ≤ 26.6	0.525 (0.445–0.619)	<0.001				
Q4 > 26.6	0.471 (0.395–0.561)	<0.001				
mBMI						
Q1 < 636.0			1			
636.0 ≤ Q2 ≤ 759.0			0.529 (0.459–0.611)	<0.001		
759.0 ≤ Q3 ≤ 884.0			0.302 (0.252–0.363)	<0.001		
Q4 > 884.0			0.207 (0.165–0.258)	<0.001		
Albumin (g/dL)						
Q1 < 2.8					1	
2.8 ≤ Q2 ≤ 3.2					0.509 (0.444–0.584)	<0.001
3.2 ≤ Q3 ≤ 3.5					0.264 (0.214–0.324)	<0.001
Q4 > 3.5					0.221 (0.172–0.282)	<0.001
APACHE II score	1.061 (1.053–1.068)	<0.001	1.056 (1.049–1.063)	<0.001	1.056 (1.049–1.063)	<0.001
Hypertension	0.931 (0.748–1.158)	0.519	0.975 (0.785–1.210)	0.819	0.945 (0.762–1.172)	0.609
Diabetes Mellitus	1.039 (0.785–1.375)	0.789	1.036 (0.785–1.367)	0.804	0.930 (0.706–1.225)	0.605
History of IHD	1.022 (0.700–1.490)	0.911	1.016 (0.697–1.481)	0.936	1.042 (0.714–1.520)	0.832
Liver Disease	1.691 (1.010–2.829)	0.046	1.376 (0.822–2.303)	0.224	1.354 (0.810–2.263)	0.248
Cancer	1.985 (1.747–2.255)	<0.001	1.920 (1.690–2.182)	<0.001	1.944 (1.710–2.209)	<0.001

cBMI, conventional body mass index; CI, confidence interval; mBMI, modified body mass index; IM, Internal Medicine; ICU, intensive care unit; APACHE II score, Acute Physiology and Chronic Health Evaluation II score; IHD, ischemic heart disease.

**Table 4:** Covariate-adjusted ROC analysis for 1-year mortality.

	Variable	AUC	*p*-Value	95% Confidence Interval	
				Lower Limit	Upper Limit
Non-adjusted	cBMI (1)	0.646	<0.001	0.628	0.664
	mBMI (2)	0.754	<0.001	0.738	0.770
	Albumin (3)	0.745	<0.001	0.730	0.761
Adjusted *	cBMI (4)	0.784	<0.001	0.769	0.798
	mBMI (5)	0.813	<0.001	0.800	0.827
	Albumin (6)	0.808	<0.001	0.794	0.822

ROC, receiver operating characteristic; AUC, area under the curve; cBMI, conventional body mass index; mBMI, modified body mass index; IM, Internal Medicine; Postop, postoperative; Adm, admission. * Covariates (gender, age, department, postoperative admission, APACHE II, Hypertension, Diabetes mellitus, history of ischemic heart disease, liver disease, and diagnosis of cancer) were adjusted to calculate the AUC. Delong’s test for two ROC curves. (1) versus (2): Z: 15.708, *p* < 0.001, (1) versus (3): Z = 8.672, *p* < 0.001, (2) versus (3): −1.474, *p* = 0.140. (4) versus (5): Z = 8.216, *p* < 0.001, (4) versus (6): Z = 4.536, *p* < 0.001, (5) versus (6): −1.653, *p* = 0.098.

## Appendix A

**Table A1:** Univariate Cox Regression Analysis for 30 day Mortality after ICU admission.

Variable	Death (*n* = 482)	Survival (*n* = 5687)	*p*-Value	Hazard Ratio (95% CI)	*p*-Value *
Sex: Male	321 (66.6%)	3398 (59.8%)	0.003	1.321 (1.093–1.596)	0.004
Age (year)	70.5 (61.0–78.0)	66.0(53.0–75.0)	<0.001	1.021 (1.015–1.028)	<0.001
Admission Department: IM	234 (48.5%)	836 (14.7%)	<0.001	4.728 (3.954–5.654)	<0.001
Postoperative Admission	318 (66.0%)	4545 (79.9%)	<0.001	0.513 (0.425–0.619)	<0.001
cBMI (kg/m^2^)	22.9(20.1–25.6)	24.2(21.8–26.6)	<0.001		
Q1 < 21.7				1	
21.7 ≤ Q2 ≤ 24.1				0.722 (0.575–0.907)	0.005
24.1 ≤ Q3 ≤ 26.6				0.473 (0.365–0.613)	<0.001
Q4 > 26.6				0.530 (0.413–0.681)	<0.001
mBMI	583.0(484.0–705.3)	24.2(21.8–26.6)	<0.001		
Q1 < 636.0				1	
636.0 ≤ Q2 ≤ 759.0				0.369 (0.296–0.460)	<0.001
759.0 ≤ Q3 ≤ 884.0				0.171 (0.127–0.231)	<0.001
Q4 > 884.0				0.120 (0.085–0.170)	<0.001
Albumin (g/dL)	2.6(2.3–2.9)	3.2(2.9–3.5)	<0.001		
Q1 < 2.8				1	
2.8 ≤ Q2 ≤ 3.2				0.266 (0.213–0.332)	<0.001
3.2 ≤ Q3 ≤ 3.5				0.115 (0.079–0.332)	<0.001
Q4 > 3.5				0.069 (0.043–0.109)	<0.001
Length of Hospital stay	24.0(12.0–42.0)	15.0(10.0–27.0)	<0.001		
Length of ICU stay	6.0(2.0–15.0)	2.0(2.0–3.0)	<0.001		
APACHE II score	28.0(22.0–34.0)	18.0(15.0–25.0)	<0.001		
Hypertension	60 (12.4%)	463 (8.1%)	0.001		
Diabetes Mellitus	31 (6.4%)	220 (3.95)	0.006		
History of IHD	12 (2.5%)	83 (1.5%)	0.078		
Liver Disease	7 (1.5%)	37 (0.7%)	0.045		
Cancer	120 (24.9%)	1214 (21.3%)	0.069		

Presented as number (percentage) or median [IQR]. IQR, interquartile range; CI, confidence interval; cBMI, conventional body mass index; mBMI, modified body mass index; ICU, intensive care unit; APACHE II score, Acute Physiology and Chronic Health Evaluation II score; IHD, ischemic heart disease.

**Table A2:** Univariate Cox Regression Analysis for 1-year Mortality after ICU admission.

Variable	Death (*n* = 1169)	Survival (*n* = 5000)	*p*-Value	Hazard Ratio (95% CI)	*p*-Value *
Sex: Male	771 (66.0%)	2948 (59.0%)	<0.001	1.306 (1.158–1.474)	<0.001
Age (year)	72.0 (62.0–79.0)	65.0 (52.0–74.0)	<0.001	1.032 (1.027–1.036)	<0.001
Admission Department: IM	448 (38.3%)	622 (12.4%)	<0.001	3.627 (3.223–4.082)	<0.001
Postoperative Admission	783 (67.0%)	4080 (81.6%)	<0.001	0.501 (0.444–0.566)	<0.001
cBMI (kg/m^2^)	22.4 (19.8–25.0)	24.4 (22.1–26.8)	<0.001		
Q1 < 21.7				1	
21.7 ≤ Q2 ≤ 24.1				0.581 (0.503–0.672)	<0.001
24.1 ≤ Q3 ≤ 26.6				0.397 (0.338–0.467)	<0.001
Q4 > 26.6				0.337 (0.284–0.401)	<0.001
mBMI	617.0 (515.5–735.0)	787.0 (674.0–904.8)	<0.001		
Q1 < 636.0				1	
636.0 ≤ Q2 ≤ 759.0				0.399 (0.347–0.459)	<0.001
759.0 ≤ Q3 ≤ 884.0				0.203 (0.170–0.242)	<0.001
Q4 > 884.0				0.124 (0.100–0.154)	<0.001
Albumin (g/dL)	2.8 (2.5–3. )	3.2 (2.9–3.6)	<0.001		
Q1 < 2.8				1	
2.8 ≤ Q2 ≤ 3.2				0.395 (0.346–0.451)	<0.001
3.2 ≤ Q3 ≤ 3.5				0.188 (0.154–0.230)	<0.001
Q4 > 3.5				0.119 (0.094–0.151)	<0.001
Length of Hospital stay	25.0 (14.0–45.0)	15.0 (10.0–25.0)	<0.001		
Length of ICU stay	3.0 (2.0–8.0)	2.0 (2.0–3.0)	<0.001		
APACHE II score	24.0 (19.0–30.0)	18.0 (15.0–24.0)	<0.001	1.072 (1.065–1.079)	<0.001
Hypertension	134 (11.5%)	389 (7.8%)	<0.001	1.465 (1.224–1.754)	<0.001
Diabetes Mellitus	76 (6.5%)	175 (3.5%)	<0.001	1.781 (1.412–2.247)	<0.001
History of IHD	29 (2.5%)	66 (1.3%)	0.004	1.732 (1.198–2.504)	0.003
Liver Disease	15 (1.3%)	29 (0.6%)	0.010	2.010 (1.207–3.344)	0.007
Cancer	380 (32.5%)	954 (19.1%)	<0.001	1.831 (1.620–2.070)	<0.001

Presented as number (percentage) or median (IQR). IQR, interquartile range; CI, confidence interval; cBMI, conventional body mass index; mBMI, modified body mass index; ICU, intensive care unit; APACHE II score, Acute Physiology and Chronic Health Evaluation II score; IHD, ischemic heart disease.
